# Characteristics of Children and Adolescents with Insomnia and Comorbid Nightmares—A Secondary Analysis of Clinical Samples with an Age Range from 0 to 18 Years

**DOI:** 10.3390/children12020129

**Published:** 2025-01-24

**Authors:** Angelika A. Schlarb, Isabel Brandhorst, Barbara Schwerdtle, Maria Zschoche, Andrea Kübler, Karolin Teichmüller

**Affiliations:** 1 Clinical Psychology and Psychotherapy for Children and Adolescents, Department of Psychology, Faculty of Psychology and Sports Science, Bielefeld University, 33615 Bielefeld, Germany; maria.zschoche@uni-bielefeld.de; 2 Department of Psychiatry, Psychosomatics and Psychotherapy in Childhood and Adolescence, University Hospital Tuebingen, 72076 Tubingen, Germany; isabel.brandhorst@med.uni-tuebingen.de; 3 Praxis Psychotherapie, 97204 Hoechberg, Germany; bs@psychotherapie-hoechberg.de; 4 Department of Psychology I, University of Würzburg, 97070 Würzburg, Germany; andrea.kuebler@uni-wuerzburg.de (A.K.); karolin.teichmueller@uni-wuerzburg.de (K.T.)

**Keywords:** insomnia, nightmare, comorbidity, emotional impairment, depression

## Abstract

Background: Insomnia disorder in childhood and adolescence has severe implications on overall well-being and development. Age-specific treatments for insomnia disorder with cognitive behavioral interventions (CBT-I) are available and effective. Nightmare disorder also has severe consequences in children and adolescents. However, less is known about children with insomnia (I) and comorbid nightmare disorder (I + N). Methods: In this retrospective study, data from 499 children and adolescents with insomnia disorder were included. The prevalence of a comorbid nightmare disorder (I + N) was calculated within three subsamples (toddlers and preschoolers 0.5–4 years, elementary school children 5–10 years, and adolescents 11–18 years). Differences between children with insomnia (I) and those with additional nightmare disorder (I + N) regarding age, sex, family background, sleep quality (SOL, WASO, TST, and SE) based on sleep logs, behavior sleep problems (based on interviews), and behavioral problems (CBCL and YSR) were calculated within each age group. Results: The overall prevalence of additional nightmares or nightmare disorder in children or adolescents with insomnia was 15–24%. We found various clinically relevant differences between I and I + N for each age group; for example, there were more sleep onset association problems in I + N elementary school children, prolonged SOL of 56 min, and about 50 min less TST and SE of 76.8% in I + N adolescents. However, most statistical tests were not significant. Especially sleep parameters but also emotional burden were more pronounced in I + N groups than in the I groups. Toddlers and preschoolers with I + N were significantly older than those with only I, had another family situation (e.g., divorced parents) significantly more often, and I + N adolescents were statistically more often anxious and depressed. Discussion: Descriptively, I + N children and adolescents seemed to be more impaired than those with insomnia only. However, a comorbid nightmare disorder cannot be recognized by insomnia-specific sleep parameters. Therefore, diagnostic procedures for insomnia should always screen for nightmares but also other sleep disorders. If necessary, CBT-I should be supplemented with nightmare-specific interventions.

## 1. Introduction

Insomnia disorder is known to be prevalent among toddlers, children, and adolescents, with significant implications for their cognitive, emotional, and physical well-being and development [1,2,3]. Insomnia includes different symptoms like difficulty initiating or maintaining sleep, early-morning awakening, or a combination thereof. Important to note is the significant stress for the children but often also for the parents and impaired functioning according to the Diagnostic and Statistical Manual of Mental Disorders [4]. Therefore, insomnia in children can take many forms, from difficulty initiating sleep to frequent night-time awakenings and unrefreshing sleep. Insomnia prevalence ranges from 20 to 30% in infants and toddlers [5,6] and up to one-third of adolescents [7,8]. Multiple severe consequences can occur, affecting, e.g., learning, behavior, and overall quality of life for the child and their family. Furthermore, insomnia does not necessarily disappear in time as children get older [9]. Even in the absence of comorbid mental health disorders, insomnia symptoms in children are associated with severe health conditions like obesity or suicidality [9,10]. When insomnia is comorbid with other mental disorders, the detrimental effects on both physical and mental health are further exacerbated [11,12].

Nightmares, which involve disturbing dreams that cause the child to wake up in distress, are another common sleep disorder. According to the DSM-5-TR, nightmares are a subgroup of parasomnia disorders involving repeated occurrences of dysphoric and well-remembered dreams that cause clinically significant distress or impairment. Nightmare frequency determines the severity of the disorder, with “mild” meaning less than once a week, “moderate” corresponding to one or more nightmares a week, and “severe” indicating nightly episodes. Symptom duration determines the chronicity of the disorder, with acute nightmares persisting for one month or less, subacute nightmares for one to six months, and persistent nightmares for six months or longer. It has to be taken into account that occasional nightmares are developmentally typical; however, chronic nightmares are often associated with other disorders and difficulties like anxiety, emotion regulation problems, stress, or traumatic events [13,14].

While precise numbers can vary depending on definitions and measurement methods [15], it is well known that nightmares affect a significant proportion of children of all ages. Nightmare frequency might peak around the age of 10, and girls tend to report more nightmares than boys between 10 and 15 years of age [15].

Insomnia and nightmares frequently co-occur in children, often exacerbating each other’s symptoms. Nightmares, especially when chronic, can lead to significant distress and impaired daily functioning. Insomnia, characterized by difficulty falling or staying asleep, is often found in children who experience frequent nightmares [16,17]. The comorbidity of insomnia and nightmares in children can significantly lower their quality of life (QoL). Children with these sleep disorders often report more difficulties in emotional and social functioning, which affects their overall well-being. Therefore, effective detection and management of these sleep disorders is crucial for improving their QoL and mental health outcomes [11]. In addition, children with mental disorders such as attention-deficit hyperactivity disorder (ADHD) are particularly vulnerable to sleep disturbances, including insomnia and nightmares. These findings emphasize the importance of early identification and further intervention for sleep disorders in children.

Behavioral therapies, especially cognitive behavioral therapy (CBT), offer effective treatment options to address both insomnia and nightmares, thereby enhancing the overall health and well-being of affected children. Previous studies mostly included mixed ages (children and adolescents) concerning the relationship between insomnia symptoms and bad dreams or nightmares and did not distinguish between insomnia and nightmare disorder and insomnia symptoms or impaired sleep quality and bad dreams [18]. However, studies evaluating the characteristics of patients with insomnia disorder and comorbid nightmares in children based on diagnostic criteria are missing. Therefore, this study will be the first to discriminate between different childhood age groups with insomnia disorder (I) and those with additional nightmare disorder (I + N). In detail, the present study aims to (1) quantify nightmare frequency in children and adolescents with insomnia, (2) characterize the subgroup with comorbid nightmares regarding demographic data (age, sex, family background, and siblings), (3) detect sleep-related differences (sleep onset latency (SOL), wake after sleep onset (WASO), total sleep time (TST), and sleep efficiency (SE)), and (4) investigate mental or emotional impairment. We hypothesized that children diagnosed with insomnia and comorbid nightmare disorder (I + N) are older and show more emotional problems and sleep impairments than those with insomnia only (I).

## 2. Methods

The present study is a secondary analysis of data from three different studies on sleep interventions for childhood insomnia in different age groups: parental sleep training for infants and toddlers between 0 and 4 years [19], a CBT-I-based intervention for children between 5 and 10 years [20], and CBT-I-based training for adolescents aged 11 to 18 years [21]. These three out-patient group interventions were conducted at the authors’ institutions in Würzburg, Bielefeld, and Tübingen between 2011 and 2020. All participants had to be diagnosed with insomnia according to the ICSD-2 or ICSD-3 and also according to ICD-10 and DSM-4 criteria. The diagnostic procedure was conducted by trained clinical psychologists under AS’s supervision with the below-mentioned instruments (Table 1). For evaluation purposes, the treatments were accompanied by comprehensive data collection, including questionnaires and sleep logs at pre- and several post-measurement points (not reported here). For detailed descriptions of the CBT-I-based intervention programs and their pre-, post-, and long-term effectiveness, see [22,23] for the youngest age-group up to four years, [24] for children aged five to ten years, and [25] for adolescents eleven years and older. For all of these previous projects, ethical permission was given by the local ethical review board (Tübingen University Approval Code: 414_2012BO1; Bielefeld University Approval Code: 2020-162; Bielefeld University Approval Code:2017-036).

For the present study, only data from the baseline measurement before treatment were included in the analysis. These data were assessed in daily routine. Children taking medication affecting sleep were excluded. Furthermore, children with severe neurological disorders, for example epilepsy, were also excluded. Because of the large age range, different age-appropriate instruments were used, and the source of information (parents’ reports or children’s/adolescents’ self-reports) also varied. Table 1 provides an overview of the assessment tools categorized by age group. Diagnoses were identified in clinical interviews or questionnaires according to the ICSD-2 or ICSD-3 (and also ICD-10 and DSM-4) criteria. Sleep parameters were calculated based on sleep log data, and behavioral and emotional problems were reported by the parents with the Child Behavior Checklist (CBCL). The adolescent sample also completed the Youth Self-Report (YSR) as a self-report measure of behavioral and emotional problems. For young children, parents were the source of information, whereas adolescents also filled in questionnaires or were interviewed by a clinical expert in addition to the parental report (see Table 1).

### Statistics

In the toddler sample, we tested associations of age, sex, family background, and siblings with parental reports of nightmare frequency, respectively, via non-parametric measures (Spearman’s rho for age, otherwise Mann–Whitney U-test). In the other groups (school-aged children and adolescents), we analyzed group differences between those diagnosed with insomnia only (I) and those with a comorbid nightmare disorder (I + N) using unpaired *t*-tests or Welch tests, if the assumption of equal variances was violated. Group differences on the CBCL and YSR were analyzed non-parametrically with Mann–Whitney U-tests, because these data were available only from smaller subsamples due to missing data. Two-tailed *p*-values were considered statistically significant for *p* < 0.05 and marginally significant for *p* < 0.10.

## 3. Results

In total, 499 children and adolescents diagnosed with insomnia disorder were included in the analysis. Demographic characteristics of the three age groups are shown in Table 2.

### 3.1. Toddlers and Preschool Children

**Figure 1:**
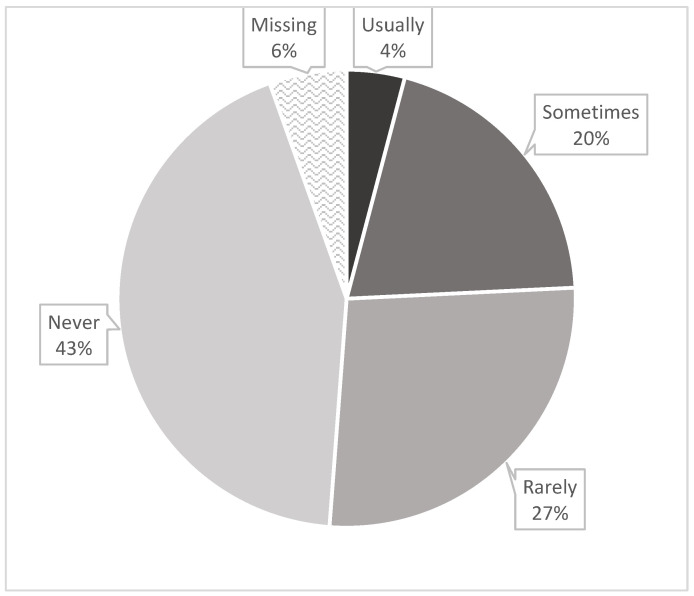
Nightmare frequency reported by parents based on questionnaire (*n* = 189; see Table 2).

All of these young children (*n* = 293; 0.5–4 years of age) fulfilled ICSD-2 or ICSD-3 criteria of insomnia disorder with daytime impairment. Overall, 4.1% of the parents stated that it was usual for their child to have nightmares, 20% reported that their children sometimes had nightmares, and 70% described nightmares rarely or not at all (Figure 1), resulting in 24% in total for children with I + N.

Older age correlated moderately significantly with more frequent nightmares (Spearman’s rho = 0.319, *p* < 0.001, *n* = 189). In this age group, there was a trend for nightmare frequency to be marginally higher in girls than in boys (see Table 3). Family background was significantly associated with parents’ reports of nightmare frequency, with children living with both parents having less nightmares compared to children living in other family situations; children with siblings did not differ from single children.

### 3.2. Elementary School Children

In this subsample (*n* = 147), a clinical nightmare disorder was prevalent in *n* = 23 (15.6%) children diagnosed with insomnia (I + N), while *n* = 118 (80.3%) children had no comorbid nightmare disorder (*n* = 6, 4.1% missing). There was no significant effect of sex, but there was a statistical trend for age. Children with I + N were on average 8 months younger than children with insomnia only (I). In contrast to the younger age group, family background and the presence of siblings were unrelated to the prevalence of a nightmare disorder in elementary school age. Qualitative results show that sleep onset latency (SOL) was above the clinical cut-off of 30 min in children with insomnia only (I), whereas the I + N group had less than 30 min. Both groups had a similar TST of about 9.8 h. However, sleep log data revealed no significant group differences in WASO, TST, or SE. SOL tended to be even shorter in the I + N group. No significant differences were found for parent-reported behavioral problems according to the CBCL (see Table 4).

**Figure 2:**
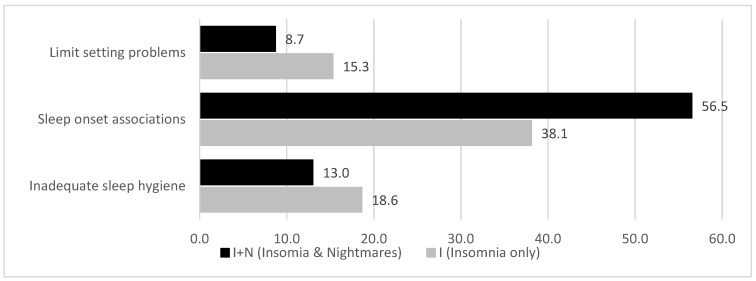
Prevalence of specific sleep-related problems in elementary school children (5–10 years) with vs. without comorbid nightmare disorder (%).

Descriptively, the most frequent sleep-related problem in children with a comorbid nightmare disorder (I + N) was sleep onset associations, whereas inadequate sleep hygiene and limit setting problems were more prevalent in children with insomnia disorder only (I) (Figure 2). However, the differences in the distributions of sleep-related problems between the I and I + N groups were not statistically significant (χ^2^(2) = 2.28, ns.).

### 3.3. Adolescents

In the adolescent subsample (*n* = 59), a nightmare disorder was diagnosed in *n* = 9 (15.3%). No sex and age differences were found. Although a higher share of females compared to males were affected by both insomnia and nightmare disorder, the effect for sex was non-significant. Both groups showed no difference in mean age either. Like in the schoolchildren subsample, family background and the presence of siblings were unrelated to the prevalence of a nightmare disorder (Table 5).

Descriptively, all sleep log parameters appeared to be more problematic in the I + N group. In detail, SOL was 16.65 min longer, WASO was 3.91 min longer, TST was 48.35 min less (and in sum an average of about 7 h), and sleep efficiency was 9.66% less at 76.8%. However, on a statistical basis, only the sleep log parameters SOL and SE differed marginally significantly between the groups.

No differences emerged for parent-reported behavioral problems and for most scales of the self-report of behavioral problems. On the subscale level, adolescents with I + N reported significantly higher scores for anxious/depressive symptoms. Interestingly, the anxious/depressive symptom subscale was the only scale on which self- and parental ratings correlated moderately (Spearman’s rho = 0.433), while all other CBCL/YSR scales showed high concurrence between self- and parental ratings, with Spearman’s rho ranging between 0.622 (internalizing problems) and 0.735 (externalizing problems).

**Figure 3:**
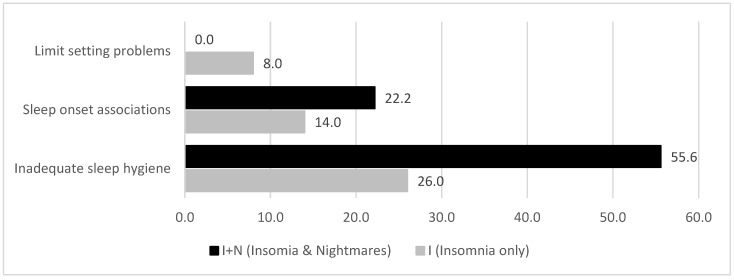
Prevalence of specific sleep-related problems in adolescents (11–18 years) with and without comorbid nightmare disorder (%).

As shown in Figure 3, sleep onset associations were more prevalent in adolescents with I + N compared to adolescents with insomnia only, although the overall prevalence of this sleep problem was lower than in the schoolchildren subsample (Figure 3). Likewise, limit setting problems were less prevalent. Many adolescents with insomnia presented with inadequate sleep hygiene, especially those with a comorbid nightmare disorder. Despite these descriptive differences, the statistical difference in the distributions of sleep-related problems between the I and I + N groups was non-significant (χ^2^(2) = 1.45, ns.).

## 4. Discussion

To summarize, a proportion of about 15% of children and adolescents diagnosed with insomnia had a comorbid nightmare disorder. For toddlers and preschool children, 24% of parents reported frequent nightmares in their child. In toddlers and elementary school children, we found slight age differences: I + N was more prevalent with older age in the toddlers and with younger age in the elementary school children. These results are in contrast to Gauchat et al., who found a peak of nightmares around the age of 10, and girls tend to report more nightmares than boys between 10 and 15 years old [15]. However, we found equal percentages of I + N in the schoolchildren and adolescents with no gender differences. Only in the youngest age group did girls tend to be affected more frequently. However, one might assume that having insomnia disorder might be a risk factor for also developing a nightmare disorder. But in their longitudinal study over two years with children of about 4 years at the baseline, Steinsbekk and Wichstrom [27] found that children with insomnia at age four were not at increased risk of being diagnosed with a nightmare disorder at age six (based on parental interviews). Furthermore, children with a nightmare disorder at age four were not at increased risk of being diagnosed with insomnia at age six [27]. Our results are also partially in contrast to a study with children of about 7.5 years of age addressing insomnia and comorbid disorders. Blader and colleagues reported that having nightmares more than twice per week was positively associated with difficulties falling asleep [28], whereas in our sample of children with I + N, we found no elevated sleep onset latency (SOL) but more sleep onset association problems reported by the parents. However, the results of Blader et al. were based on a community survey (parent reports) without a clinical interview or any standardized sleep questionnaire. A further study with slightly older children (9.2 years of age) found a positive relationship between each of four insomnia symptoms (difficulty initiating sleep, difficulty maintaining sleep, early-morning awakening, and restless sleep) and frequency of nightmares [16]. Thus, parts of our results are in line with these as we also found that sleep onset associations were elevated in this age group. However, concerning SOL, we found opposite effects, with children with I + N having less SOL than I only (statistically and qualitatively). Methodologically, Li and colleagues’ study also implemented a semi-structured clinical interview for DSM-IV but no specialized sleep disorder interview or sleep log [16].

In our study, adolescents with I + N had clinically severe sleep quality impairments compared to those with I only. In addition, insomnia symptoms were slightly increased in adolescence if a comorbid nightmare disorder was present. Sleep efficiency was 76.8%, which is under the recommended percentage of 85% and therefore clinically highly relevant. The prevalence of limit setting problems and sleep onset associations was lower, while inadequate sleep hygiene occurred more often than in the children’s sample. This may be due to adolescents becoming increasingly independent of their parents in managing their bedtime routine. A study conducted by Russel and colleagues [29] with adolescents of 15 years of age found that nightmare severity was positively associated with the severity of insomnia symptoms. Therefore, our results seem partly in line with those of Russel and colleagues. However, their study was based on questionnaires only without clinical interview or sleep log data. Another study [30] also addressed adolescents (12–18 years), assessed insomnia symptoms with the Insomnia Severity Index (ISI), and found that insomnia severity and depression symptoms mediated the positive association between nightmare distress and cognitive deficits. Our results seem to be in line with those as we also found differences concerning anxiety and depression of adolescents with I + N compared with those with insomnia only.

As our results also show that children and adolescents with I + N often had sleep onset associations or sleep-incompatible behaviors, which might be dysfunctional strategies for dealing with sleep- or nightmare-related fears, explaining the higher proportion of sleep onset associations and inadequate sleep hygiene in this subgroup. Therefore, concerning the diagnostic procedure of insomnia disorders in childhood and adolescence, it always has to be considered that a percentage of a minimum of 15% might also have a comorbid nightmare disorder. So, not only insomnia symptoms but also other sleep disorders such as nightmares or night terrors should be assessed if a child has difficulties falling asleep or maintaining sleep. As was stated in the stepped-care model for child and adolescent insomnia [31], clinicians and experts should pay special attention to diagnostic procedure and not overlook other disorders like nightmare disorders in children with insomnia symptoms. Our results are also in line with the review of Delage and colleagues [18], pointing to an interconnectivity between insomnia and nightmares and showing that insomnia might increase the likelihood of nightmares, and also, in turn, nightmares can lead to sleep loss and non-restorative sleep. Based on our findings, more sensitive diagnostic measurements should be developed, and future research should focus on comorbid disorders and also on family contexts. Given the retrospective and cross-sectional nature of our data, longitudinal studies might also help to understand the complex interplay between various sleep disorders at a young age. Beyond this, more detailed research concerning further sleep aspects like restorative sleep, pre-sleep arousal, or pre-sleep cognition might also help to further explore the association between insomnia and nightmares.

## 5. Strengths

We re-analyzed a large data set of 499 patients diagnosed with insomnia and comorbid nightmare disorder in three different age groups spanning from toddlers to adolescents. Whereas other studies often focus on insomnia symptoms or sleep quality exclusively, the individuals in our sample with insomnia and nightmare disorder for elementary school children and adolescents were diagnosed with a structured interview or questionnaires, and all were diagnosed based on ICSD-2 or ICSD-3 criteria. Besides the proper diagnosis, sleep data based on sleep diary protocols across two weeks were included. Potentially related variables were assessed with standardized questionnaires like the CBCL. For adolescents, self-related questionnaires were also implemented.

## 6. Limitations

As a general limitation to secondary analyses, we acknowledge that the data presented here were not primarily assessed for this study purpose. Thus, missing values and unequal sample sizes for the three age groups weaken the informative value of our results. While we found associations of nightmare frequency and demographic variables in our largest subsample, i.e., toddlers and preschool children, the elementary school children and especially the adolescent samples lack the statistical power to detect small effects. With α = 0.05 and β = 0.80, the sample size of the elementary school children had the power to detect group differences of medium effect size (d ≈ 0.5), but our adolescent sample size is sufficient for the detection of large effects (d > 0.8) only. We did not include measurements like actigraphy or polysomnography and did not diagnose other mental disorders.

## 7. Conclusions

In sum, this study included 499 children and adolescents all diagnosed with insomnia disorder. Various other facets next to nightmare disorder were assessed like daytime impairment or family aspects. On the basis of a large sample of toddlers and preschool children, elementary school children, and adolescents diagnosed with insomnia, we found comorbid nightmares or nightmare disorder in 24%, 15.6%, and 15.3% of the participants. We found clinically relevant differences between I and I + N, pointing toward more sleep impairment of patients with I + N and more mental burden. However, most sleep parameters and measures of behavioral problems did not differ statistically significant between children with insomnia with vs. without comorbid nightmares, suggesting that insomnia might mask the symptoms of a comorbid nightmare disorder. In sum, these results show that not only sleep but also mental impairments are more pronounced in those patients. Prevention programs as well as implementing early-intervention strategies for other sleep disorders like nightmares might help to prevent chronification of sleep disorders and strengthen mental health. Further, as it may cause additional distress, contributing to maintenance of insomnia, and affect daytime behavior, it is essential to specifically address nightmares when children present with insomnia symptoms. But information strategies for health care providers like pediatricians and child psychologists or therapists and also information for parents regarding possible comorbidity and the association with mental health might also be important. Children’s books should address sleep problems to teach strategies that help children to deal with these problems at a young age in a solution-oriented way. For sleep-related anxiety, the effectiveness of such books has already been demonstrated [32].

## Figures and Tables

**Table 1:** Diagnostic measures.

	Reference	Content	Applicable for Age Group	Source of Information
Toddlers and preschool children				
Anamnesis form	Developed by AS	Demographics, medical history, sleep	0–4 years	Parent
Elementary school children				
Anamnesis form	Developed by AS	Demographics, medical history, sleep	5–10 years	Parent
Structured sleep interview	Developed by AS according to ICSD-2	ICSD-2/3 criteria of sleep disorders; ICD-10 and DSM-4 criteria	5–10 years	Parent
Sleep logs		SOL, WASO, TST, SE		Parent
Child Behavior Checklist (CBCL/4–18)	Achenbach, 1991	Emotional and behavioral problems	4–18 years	Parent
Adolescents				
Anamnesis form	Developed by AS	Demographics, medical history, sleep	11–18 years	Adolescent
Structured sleep interview (parents’ version)	Developed by AS according to ICSD-2	ICSD-2/3 criteria of sleep disorders, ICD-10 and DSM-4 criteria	11–18 years	Parent
Structured sleep interview (adolescents’ version)	Developed by AS according to ICSD-2	ICSD-2/3 criteria of sleep disorders; ICD-10 and DSM-4 criteria	11–18 years	Adolescent
Sleep logs		SOL, WASO, TST, SE		Adolescent
Child Behavior Checklist (CBCL/4–18)	Achenbach, 1991	Emotional and behavioral problems	4–18 years	Parent
Youth Self-Report (YSR/11–18)	Achenbach, 1991	Emotional and behavioral problems	11–18 years	Adolescent

Note: SOL—sleep onset latency; WASO—wake after sleep onset; TST—total sleep time; SE—sleep efficiency; AS—Angelika Schlarb [26].

**Table 2:** Demographic characteristics.

	Toddlers and Preschool Children (0–4 y) *n* = 293	Elementary School Children (5–10 y) *n* = 147	Adolescents (11–18 y) *n* = 59
Age [months] (M ± SD)	22.36 (±12.60) Min 7, Max 63 92 missing	94.68 (±20.74) Min 58, Max 136 None missing	161.85 (±20.31) Min 129, Max 212 None missing
Sex *n* (%)			
Male	155 (52.9%)	75 (51.0%)	24 (40.7%)
Female	138 (47.1%)	72 (49.0%)	35 (59.3%)
Family background *n* (%)			
Lives with both parents	281 (95.9%)	123 (83.7%)	49 (83.1%)
Other family situation	11 (3.8%)	23 (15.6%)	9 (15.3%)
Not specified	1 (0.3%)	1 (0.7%)	1 (1.7%)
Siblings *n* (%)			
0	176 (60.1%)	27 (18.4%)	10 (16.9%)
1 or more	117 (36.5%)	118 (80.2%)	48 (81.4%)
Not specified	10 (3.4%)	2 (1.4%)	1 (1.7%)

**Table 3:** Associations of nightmare frequency and demographic characteristics in toddlers and preschoolers (0–4 years, *n* = 293).

	Nightmare Frequency (Mean Rank ^1^)	Group Difference
Sex		U = 8448.5, Z = −1.79, *p* = 0.073 ^ǂ^, *n* = 277
Male	131.37	
Female	147.51	
Family background		U = 838.5, Z = −2.13, *p* = 0.033 *, *n* = 276
Lives with both parents	136.65	
Other family situation	187.65	
Siblings		U = 8244.5, Z = −0.13, ns., *n* = 267
0	134.43	
1 or more	133.28	

^1^ A higher rank indicates more frequent nightmares, ^ǂ^ *p* < 0.10, * *p* < 0.05.

**Table 4:** Group differences of elementary school children with vs. without a comorbid nightmare disorder (5–10 years, *n* = 147).

	I (*n* = 118, 80.3%)	I + N (*n* = 23, 15.6%)	Group Difference
Age [months] M (SD)	95.83 (20.79)	87.65 (20.03)	*t*(139) = 1.74, *p* = 0.085 ^ǂ^, *n* = 141
Sex *n* (%)			Chi2(1) = 0.07, ns., *n* = 141
Male	60 (50.8%)	11 (47.8%)	
Female	58 (49.2%)	12 (52.2%)	
Family background *n* (%)			Chi2(1) = 0.86, ns., *n* = 140
Lives with both parents	98 (83.8%)	21 (91.3%)	
Other family situation	19 (16.2%)	2 (8.7%)	
Siblings *n* (%)			Chi2(1) = 0.07, ns., *n* = 139
0	21 (18.1%)	4 (17.4%)	
1 or more	95 (81.9%)	19 (82.6%)	
Sleep parameter M (SD)			
SOL	36.72 (20.87)	27.54 (14.76)	*t*(25.08) = 1.96, *p* = 0.061 ^ǂ^, *n* = 82
WASO	6.74 (9.13)	7.85 (9.21)	*t*(80) = −0.44, ns., *n* = 82
TST	587.78 (40.55)	590.69 (22.14)	*t*(80) = −0.26, ns., *n* = 82
SE	93.13 (2.98)	94.45 (2.66)	*t*(80) = −1.53, ns., *n* = 82
Behavioral problems ^1^ Parent report CBCL M (SD)			
Total	33.17 (21.47)	34.43 (20.81)	*U* = 85.5, *Z* = −0.76, ns. *n* = 37
Internalizing	9.93 (6.86)	11.00 (5.07)	*U* = 96.5, *Z* = −0.33, ns. *n* = 37
Externalizing	10.50 (8.10)	11.86 (8.40)	*U* = 98.0, *Z* = −0.27, ns. *n* = 37
Anxious/Depressed	5.67 (4.77)	6.57 (4.47)	*U* = 85.0, *Z* = −0.78, ns. *n* = 37
Attention Problems	4.40 (4.00)	4.29 (4.23)	*U* = 105.0, *Z* < 0.01, ns. *n* = 37
Aggressive Behavior	8.50 (6.74)	10.29 (7.87)	*U* = 92.5, *Z* = −0.49, ns. *n* = 37

^ǂ^ *p* < 0.10. ^1^ CBCL data were only available from subsets of *n* = 37 (*n* = 7 I + N). Group comparisons were therefore conducted with the non-parametric U-test. SOL: sleep onset latency; WASO: wake after sleep onset; TST: total sleep time (all in minutes); SE: sleep efficiency; CBCL: Child Behavior Checklist.

**Table 5:** Group differences of adolescents with and without a comorbid nightmare disorder (11–18 years, *n* = 59).

	I (*n* = 50, 84.7%)	I + N (*n* = 9, 15.3%)	Group Difference
Age [months] M (SD)	161.8 (20.03)	162.11 (23.11)	*t*(57) = −0.04, ns., *n* = 59
Sex *n* (%)			Chi2(1) = 1.77, ns., *n* = 59
Male	23 (46.0%)	2 (22.2%)	
Female	27 (54.0%)	7 (77.8%)	
Family background *n* (%)			Chi2(1) = 0.37, ns., *n* = 58
Lives with both parents	42 (85.7%)	7 (77.8%)	
Other family situation	7 (14.3%)	2 (22.2%)	
Siblings *n* (%)			Chi2(1) = 0.19, ns., *n* = 58
0	8 (16.35)	2 (22.2%)	
1 or more	41 (83.7%)	7 (77.8%)	
Sleep parameter M (SD)			
SOL	39.75 (30.83)	56.40 (41.33)	*t*(49) = −1.38, *p* = 0.087 ^ǂ^, *n* = 51
WASO	8.66 (16.50)	12.57 (30.74)	*t*(49) = −0.55, ns., *n* = 51
TST	477.08 (52.21)	428.73 (107.97)	*t*(8.86) = 1.31, ns., *n* = 49
SE	86.46 (7.96)	76.80 (17.55)	*t*(8.64) = 1.62, *p* = 0.071 ^ǂ^, *n* = 56
Behavioral problems ^1^ Parent report CBCL M (SD)			
Total	33.71 (22.18)	31.80 (16.10)	*U* = 41.0, *Z* = −0.12, ns. *n* = 22
Internalizing	11.24 (7.62)	12.40 (9.94)	*U* = 42.0, *Z* = −0.04, ns. *n* = 22
Externalizing	8.71 (7.25)	7.00 (5.24)	*U* = 40.0, *Z* = −0.20, ns. *n* = 22
Anxious/Depressed	4.59 (4.26)	5.20 (4.87)	*U* = 40.5, *Z* = −0.16, ns. *n* = 22
Attention Problems	4.18 (4.20)	3.80 (3.03)	*U* = 42.5, *Z* < 0.01, ns. *n* = 22
Aggressive Behavior	6.71 (4.96)	5.00 (4.95)	*U* = 34.5, *Z* = −0.63, ns. *n* = 22
Behavioral problems ^1^ Self-report YSR M (SD)			
Total	38.06 (16.85)	47.60 (11.48)	*U* = 25.5, *Z* = −1.20, ns. *n* = 21
Internalizing	12.31 (7.41)	18.20 (8.47)	*U* = 24.0, *Z* = −1.33, ns. *n* = 21
Externalizing	10.19 (5.61)	9.40 (7.70)	*U* = 34.5, *Z* = −0.46, ns. *n* = 21
Anxious/Depressed	4.88 (3.88)	10.00 (4.58)	*U* = 14.0, *Z* = −2.18, *p* = 0.029 *, *n* = 21
Attention Problems	4.56 (2.19)	5.00 (2.24)	*U* = 35.0, *Z* = −0.42, ns. *n* = 21
Aggressive Behavior	7.63 (4.49)	6.40 (5.59)	*U* = 31.5, *Z* = −0.71, ns. *n* = 21

^ǂ^ *p* < 0.10, * *p* < 0.05. ^1^ CBCL and YSR data were only available from subsets of *n* = 22 and *n* = 21 participants, respectively (*n* = 5 I + N). Group comparisons were therefore conducted with the non-parametric U-test. SOL: sleep onset latency; WASO: wake after sleep onset; TST: total sleep time; SE: sleep efficiency; CBCL: Child Behavior Checklist; YSR: Youth Self-Report.

## Data Availability

Data are available on request from the first author. The data are not publicly available due to privacy.
